# Newly Diagnosed Multifocal GBM: A Monocentric Experience and Literature Review

**DOI:** 10.3390/curroncol29050280

**Published:** 2022-05-11

**Authors:** Valentina Baro, Giulia Cerretti, Michela Todoverto, Alessandro Della Puppa, Franco Chioffi, Francesco Volpin, Francesco Causin, Fabio Busato, Pasquale Fiduccia, Andrea Landi, Domenico d’Avella, Vittorina Zagonel, Luca Denaro, Giuseppe Lombardi

**Affiliations:** 1 Academic Neurosurgery, Department of Neurosciences, University of Padova, 35128 Padova, Italy; michela.todoverto@aopd.veneto.it (M.T.); andrea.landi@unipd.it (A.L.); domenico.davella@unipd.it (D.d.); luca.denaro@unipd.it (L.D.); 2 Department of Oncology, Oncology 1, Veneto Institute of Oncology IOV-IRCCS, 35128 Padua, Italy; giulia.cerretti@iov.veneto.it (G.C.); vittorina.zagonel@iov.veneto.it (V.Z.); giuseppe.lombardi@iov.veneto.it (G.L.); 3 Neurosurgery Unit, Azienda Ospedaliera Universitaria Careggi, 50134 Firenze, Italy; alessandro.dellapuppa@unifi.it; 4 Neurosurgery Unit, Azienda Ospedale-Università di Padova, 35128 Padova, Italy; franco.chioffi@aopd.veneto.it (F.C.); francesco.volpin@aopd.veneto.it (F.V.); 5 Neuroradiology Unit, Azienda Ospedale-Università di Padova, 35128 Padova, Italy; francesco.causin@aopd.veneto.it; 6 Radiotherapy Unit, Veneto Institute of Oncology IOV-IRCCS, 35128 Padua, Italy; fabio.busato@iov.veneto.it; 7 Clinical Research Unit, Veneto Institute of Oncology IOV-IRCCS, 35128 Padua, Italy; pasquale.fiduccia@iov.veneto.it

**Keywords:** multifocal, multicentric, glioblastoma, survival, oncology, surgery

## Abstract

**Simple Summary:**

Glioblastoma is an aggressive brain tumor with a dismal prognosis. In a minority of cases, it presents with multiple lesions already at the time of diagnosis, affecting patients’ survival and treatment. Our retrospective study aims to increase the current understanding and define a treatment for this sub-entity, to improve patient survival. Chemoradiotherapy is a also safe and efficacy treatment in patients with multiple lesions. Survival advantages from extensive resection remain unclear.

**Abstract:**

Glioblastomas with multiple foci at presentation (mGBMs) account for 2–35% of all GBMs. mGBMs have limited existing data and no standardized treatment. This study aims to determine their incidence, demographic and clinical features, outcome, and prognostic factors in terms of overall survival. We performed a monocentric retrospective study, reviewing patients treated at the Istituto Oncologico Veneto. Inclusion criteria were: new diagnosis of GBM and presence of multiple lesions on pre-treatment MRI. ECOG PS was used to evaluate clinical condition, RANO criteria for radiological assessment, and CTCAE v5.0 for treatment-related adverse events. The incidence of newly diagnosed mGBM was 7.2% and the study population consisted of 98 patients. Median age was 63 years, M:F ratio of 1.8:1, and a surgical approach was undertaken in 73 patients (mostly partial resection). MGMT was methylated in 47.5%, and 82 patients received active oncological treatment (65.9% radiotherapy plus temozolomide (RT + TMZ)). The disease control rate with RT + TMZ was 63%. Median OS of the entire study population was 10.2 months (95% CI 6.6–13.8), and median PFS was 4.2 months (95% CI 3.2–5.2). The ECOG PS, the extent of resection, and the RT + TMZ were significant prognostic factors in the univariate analysis for OS, but only the RT + TMZ was a significant independent OS predictor in the multivariate analysis (HR = 3.1, 95% IC 1.3–7.7, *p* = 0.014). The incidence of mGBM is not rare. RT + TMZ is confirmed to be an independent prognostic factor for survival and a safe and effective treatment. When feasible, RT + TMZ should be considered as a possible first-line treatment. The role of the extent of resection is still unclear.

## 1. Introduction

Glioblastomas (GBMs) are the most common and aggressive primary malignant CNS tumors, with an incidence rate of 3.23 per 100,000 population (age-adjusted to the 2000 US standard population) [1]. They are characterized by a dismal prognosis with a median survival of 14.6 months after optimal treatment consisting of maximal safe resection followed by radiotherapy plus concomitant and adjuvant temozolomide (RT + TMZ) [2]. Established prognostic factors are the extent of resection (EOR), performance status (PS) at diagnosis, histological features, patient age, and molecular biomarker status (mutations in isocitrate dehydrogenase 1, IDH1, and promoter methylation of O6-methylguanine DNA methyltransferase, MGMT) [3,4]. Practically, all patients show disease progression after first-line treatment and the median progression-free survival (PFS) is seven months [2].

GBMs usually occur as a solitary lesion, but in a minority of cases, they present with multiple lesions (mGBM) at diagnosis, with a reported incidence of 2–35% [5,6,7,8,9,10,11,12,13,14,15]. In 1963, Batzdorf and Malamud distinguished the mGBMs into two categories based on communication between the lesions: multifocal and multicentric GBMs [16]. Multifocal GBMs have a macroscopic and/or microscopic connection among lesions. In contrast, multicentric GBMs are characterized by completely separated foci without a clear route of connection. The pathogenesis of the latter is less clear. One hypothesis is the two-step tumor process in which in the first step a large area or the entire brain is exposed to neoplastic transformation (initiation) evolving into a condition more prone to tumor development. In the second step (promotion), different triggers (i.e., biochemical, hormonal, mechanical, environmental, or viral factors) stimulate the neoplastic proliferation in multiple distinct sites. Another theory is the existence of ways of diffusion not yet known [5]. However, thanks to advanced modern imaging, true multicenter GBMs are very rare [14]. Indeed, several studies displayed no clinical relevance in distinguishing between multicentric and multifocal GBM, because of their same clinical and prognostic features [17,18,19]. Patients with multiple foci at presentation are particularly difficult to treat, carrying a worse outcome [20] due to a wider dissemination, a more common implication of eloquent and/or deep cerebral areas, a more frequent presence of an unfavorable PS, a limited surgical removal, and a higher heterogeneity rather than to the multiplicity itself [8,14]. Furthermore, the management of these patients is controversial and not yet standardized in the literature. While histopathological diagnosis is deemed necessary, the type of surgical approach is still questionable. Some studies promote an aggressive surgical treatment with maximum safe resection to obtain a longer survival, assuming adjuvant therapy to be more effective after tumor debulking [15]. On the other hand, some studies favor the role of biopsy, arguing that extensive resection could increase the comorbidities without improving survival [11]. Moreover, after the biopsy or resection, there is no guideline for radiotherapy and chemotherapy, and some wonder whether it is useful to undertake any active oncological treatment rather than palliative care alone. TMZ also remains the first-choice chemotherapy treatment in mGBM. Long-term TMZ administration after the standard therapy might be a possibility, particularly when deep-seated structures are involved [21] and, because of the diffuse disease and the microscopic dissemination of mGBMs, whole-brain radiotherapy (WBRT) should be considered [18].

Despite the advancement of neurosurgical techniques and neurooncological therapies, encouraging outcomes of patients with mGBM are not essentially observed. Few articles have explored this issue so far and their results are not comparable [6,7,8,9,10,11,12,13,14,15,17,18]. Therefore, the purpose of this retrospective mono-institutional study is to assess the demographic characteristics, the incidence, the overall survival (OS), the progression-free survival (PFS), and the prognostic factors for survival (i.e., clinical features, extent of resection, molecular characteristics, oncological treatment) in patients with newly diagnosed mGBM. Furthermore, the best response to treatment and safety are analyzed. These data could expand the current knowledge of the disease and improve patients’ management, increasing their survival and QoL.

## 2. Materials and Methods

This is a retrospective monocentric study involving the Istituto Oncologico Veneto (IOV) and the University Hospital of Padova (Azienda Ospedale-Università di Padova, AOUP). All patients with mGBM at the time of diagnosis and treated at the IOV between January 2011 and March 2021 were considered. Patients were selected from the IOV brain tumor registry database, searching the following keywords: “glioblastoma”, “high-grade glioma”, and “multifocal”. The systematic retrospective collection of patients’ data was carried out through a computerized electronic medical system containing patient medical records.

The inclusion criteria for this study were: new histological diagnosis of glioblastoma according to the World Health Organization (WHO) classification of brain tumors 2021 [22] or highly suspected neuro-radiological diagnosis for patients who did not undergo surgery (patients without histological diagnosis had highly suggestive imaging for GBM and were also subjected to further in-depth imaging, such as PET-MRI, PET-CT, and MRI spectroscopy); presence of multiple lesions on pre-treatment magnetic resonance imaging (MRI). Patients with multiple lesions were defined as those having at least 2 separate foci of enhancing tumor on MRI, separated by at least 1 cm. Glioblastoma patients with single lesion or doubt for the presence of multiple lesions at neuroradiological review or with only one hospital consultation (very short follow-up) were excluded.

A new detailed database was created containing anamnestic, epidemiological, and clinical information, details of the surgical approach, histopathological characterization, and treatment strategy.

To evaluate the clinical condition at the time of diagnosis, the WHO performance status called also the Eastern Cooperative Oncology Group (ECOG) score was applied. The EOR was stratified with the regard of pre-operative and post-operative imaging (MRI) as gross total resection (GTR), (all the enhancing tumor resected), partial resection (PR) (<100%–>10%), and biopsy (open biopsy or stereotactic biopsy) (≤10%). This stratification was made on the basis of cut-offs used in the literature, specifically the studies of Patil et al. [8] and Paulsson et al. [11].

MGMT promoter methylation status was performed by the methylation-specific PCR method or by pyrosequencing (a cut-off of 9% of methylation was applied). IDH1–2 mutation status was determined using immunohistochemistry and/or direct DNA sequencing.

The Response Assessment in Neuro-Oncology (RANO) criteria were adopted to evaluate the therapeutic response to treatment. Usually, MRI assessment was done before starting the oncological treatment and every two months or when clinically indicated. The overall response rate (ORR) was defined as the quota of patients who had a partial or complete response to therapy (PR + CR). The disease control rate was described as the portion of patients who had achieved at least a stable disease (CR + PR + SD). Progression was defined through the RANO criteria as a ≥25% increase in contrast-enhancing burden, presence of new lesions, or symptomatic progression. If pseudoprogression was clinically suspected, new imaging was acquired at a time interval to confirm the suspicion. If progression was established, the initial MRI was considered as the date of progression. In the statistical analysis of this study, the best response to treatment was considered as the best response recorded from the start of the treatment until the disease progression. Treatment-related toxicities were classified according to the Common Terminology Criteria for Adverse Events (CTCAE) version 5.0, according to the standardized definitions for adverse events (AEs) published by The National Cancer Institute (NCI) of the National Institutes of Health (NIH).

PFS was calculated as the time in months from the date of diagnosis to the date of progression, last available follow-up, or death. OS was defined as the time in months from the date of diagnosis to the date of death or last available follow-up. Censored patients were those who had not experienced the event or were lost to follow-up during the observation period. Variables analyzed to identify a possible impact on survival were the extent of resection (PR vs. biopsy), the ECOG PS (0–2 vs. >2), the MGMT status (methylated vs. unmethylated), the active oncological treatment (yes vs. no), and the RT + TMZ (yes vs. no).

Statistical analyses were performed by SPSS v22 software.

The Kaplan–Meier method was used to plot survival curves of OS and PFS. The curves were compared using the univariate log-rank test giving the statistical significance and evaluating the influence of cofactors on OS and PFS. Cox proportional hazard regression models were used for multivariate analysis to analyze the effect of independent prognostic factors on OS. A backward elimination approach was applied to derive a final multivariate Cox proportional hazard model. A *p*-value < 0.05 was defined as statistically significant. Results were presented with 95% confidence intervals (95% CI).

## 3. Results

The IOV brain tumor registry database searched using the above-mentioned keywords yielded 1354 patients with a diagnosis of glioblastoma. Hence, 98 patients met both the inclusion and exclusion criteria and were considered for the analysis as mGBM accounting for 7.2% of all patients affected by GBM treated during the same period.

**Figure 1:**
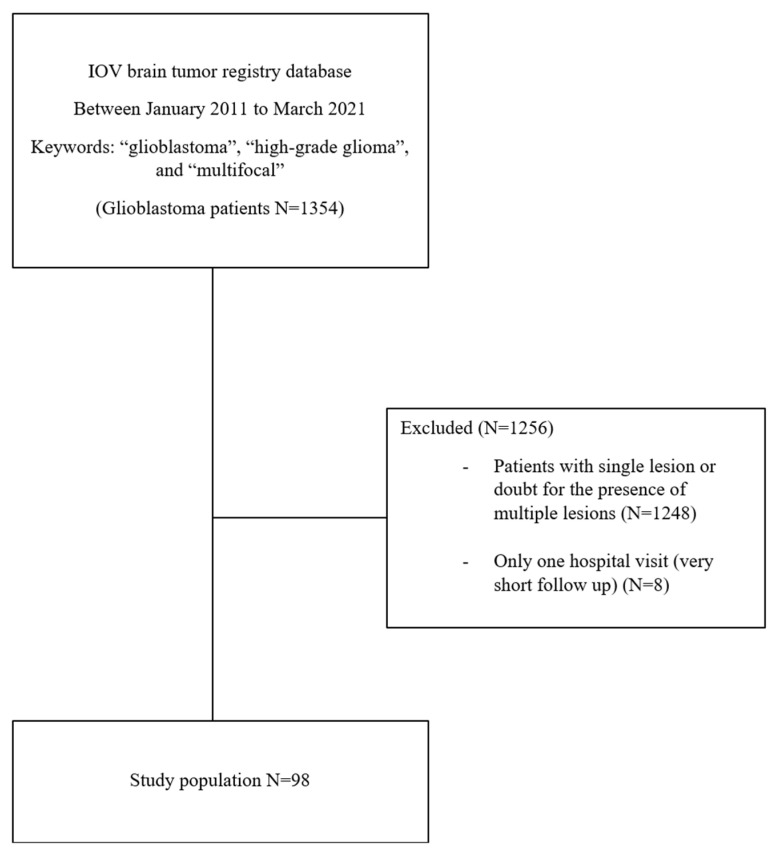
Summary of the patient selection process.

A complete flow diagram of patient selection is provided in Figure 1.

### 3.1. Patient Characteristics

Epidemiological and clinical features, molecular findings, and treatment information of patients are shown in Table 1.

In our series, the median age at diagnosis was 63 years (range 16–84). M:F ratio was 1.8:1. ECOG PS was between 0 and 2 in 74 patients (75.5%), higher than 2 in 15 patients (15.3%), and it was not available in nine subjects (9.2%).

Seventy-three (74.5%) patients underwent surgery allowing a histological diagnosis. A PR was performed in 45 (61.6%) and a biopsy in 26 (35.6%) patients. Surgical approach was not otherwise specified in the two remaining cases (2.7%). Regarding molecular markers, MGMT promoter methylation status and IDH mutation were analyzed in the 73 patients with histological diagnosis. Data regarding the MGMT promoter methylation status was available in 59 patients (80.8%) and in 28 out of 59 cases (47.5%) it was methylated. IDH mutation information was available in 60 patients (82.2%) and in all these patients it was wild-type, according to the WHO classification 2021.

Eighty-two patients (83.7%) received an active oncological treatment. Instead, 16 patients (16.3%) received only palliative care (i.e., steroids, antiepileptic drugs if needed, pain killers). Among the 82 subjects treated with active oncological treatment, 54 (65.9%) received radiotherapy with temozolomide (RT + TMZ), 26 (31.7%) temozolomide alone, and two (2.4%) radiotherapy alone. The radiotherapy administered to the patients was conformal. Patients treated with temozolomide, alone or adjuvant after the concomitant radiotherapy, received a median of two cycles (range 0–16). The reason for the treatment discontinuation was disease progression.

### 3.2. Response to Treatment

Considering the 82 patients receiving active oncological treatments, six (7%) showed a partial response (PR), 38 (46%) had a stable disease (SD), and 38 (46%) showed a progressive disease (PD). Consequently, the overall response rate (ORR = CR + PR) was 7% and disease control rate (DCR = CR + PR + SD) was 54% (Table 2).

In particular, the best response to treatment was obtained with RT + TMZ. Out of these patients, four (7%) showed a PR, 30 (56%) a SD, and 20 (37%) a PD. Therefore, ORR was 7% and DCR 63%. Instead, DCR with temozolomide alone was 38% and with radiotherapy alone was 0%.

### 3.3. Progression and Survival

**Figure 2:**
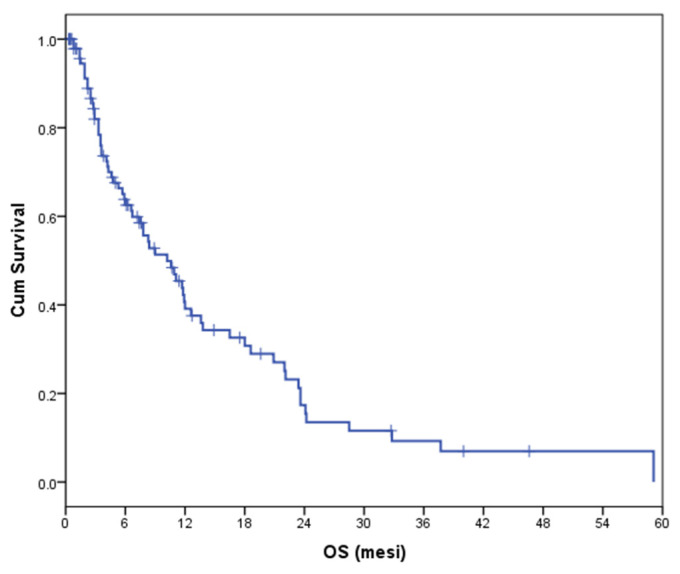
Overall survival of the study population (median OS = 10.2 months).

At the time of the analyses, 66 patients (67%) had died and median OS was 10.2 months (95% CI 6.6–13.8) (Figure 2). Hence, 83 patients (85%) progressed and median PFS was 4.2 months (95% CI 3.2–5.2) (Figure S1). The median follow-up was 6.1 months.

#### 3.3.1. Overall Survival 

**Figure 3:**
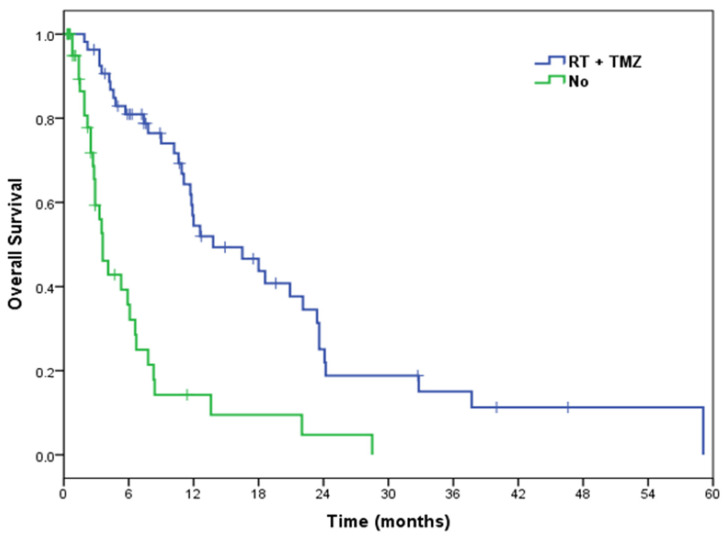
OS Kaplan-Meier curves for the RT + TMZ: yes (median OS = 13.8 months) vs. no (median OS = 3.6 months).

On univariate analysis, patients treated with PR had a median OS of 13.8 months (95% CI 6.0–21.6) and those patients who underwent biopsy 10.2 months (95% CI 5.5–14.9) (*p* = 0.028) (Figure S2). Subjects with ECOG PS 0–2 had a median OS of 11.7 months (95% CI 9.9–13.5) and those with ECOG PS >2 of 3.5 months (95% CI 3.1–3.9) (*p* = 0.012) (Figure S3). Patients with methylated MGMT promoter had a median OS of 20.9 months (95% CI 6.4–35.4) and patients with unmethylated MGMT promoter 12.6 months (95% CI 10.0–15.2) (*p* = 0.138) (Figure S4). Patients who received active oncological treatment showed a median OS of 11.1 months (95% CI 8.2–14.0) and patients who had not received it 1.9 months (95% CI 0.7–3.1) (*p* < 0.001) (Figure S5). Subjects receiving TMZ + RT had a median OS of 13.8 months (95% CI 6.9–20.7) and those who had not received RT + TMZ 3.6 months (95% CI 2.6–4.7) (*p* < 0.001) (Table 3) (Figure 3).

The multivariate OS analysis with Cox regression showed that the RT + TMZ (no vs. yes) was an independent prognostic factor (HR = 3.1, 95% CI 1.3–7.7, *p* = 0.014). Conversely, the ECOG PS (HR = 3.0, 95% CI 0.9–9.6, *p* = 0.070) and the MGMT status (HR = 2.1, 95% CI 0.9–5.0, *p* = 0.075) did not reach statistical significance (Table 4).

In summary, the univariate analysis demonstrated that patients with ECOG PS 0–2, patients undergoing PR, patients receiving active oncological treatment, and patients treated with RT + TMZ showed statistically significantly improved OS. However, only the RT + TMZ was an independent OS predictor in the multivariate analysis. Patients who did not undergo RT + TMZ had a three-fold greater risk of death compared with those treated with RT + TMZ.

#### 3.3.2. Progression-Free Survival 

On univariate analysis, patients with PR had a median PFS of 6.6 months (95% CI 5.0–8.2), and patients who underwent biopsy 3.6 months (95% CI 2.6–4.6) (*p* = 0.007) (Figure S6). Patients presenting ECOG PS 0–2 had a median PFS of 4.8 months (95% CI 3.5–6.1) and those with ECOG PS >2 3.3 months (95% CI 2.0–4.6) (*p* = 0.331) (Figure S7). Patients with methylated MGMT promoter had a median PFS of 5.4 months (95% CI 2.2–8.6), and patients with unmethylated MGMT promoter 6.8 months (95% CI 5.0–8.6) (*p* = 0.137) (Figure S8). Patients who received active oncological treatment showed a median PFS of 4.8 months (95% CI 3.7–5.9), and patients without any oncological treatment reported a PFS of 1.7 months (95% CI 1.1–2.3) (*p* < 0.001) (Figure S9). Subjects receiving RT + TMZ had a median PFS of 6.9 months (95% CI 4.9–8.9), and patients who had not received RT + TMZ 2.4 months (95% CI 1.9–2.9) (*p* < 0.001) (Table S1) (Figure S10).

In summary, significant predictors of reduced PFS were the biopsy, the absence of active oncological treatment, and the absence of RT + TMZ.

### 3.4. Safety

The most common GBM treatment-related toxicity was the heamatological toxicity, in particular thrombocytopenia and anemia, and mainly with a mild grade of severity ). Regarding haematological toxicity, 21% of 54 patients treated with RT + TMZ presented anemia (one grade 3–4), 6% neutropenia (one grade 3–4), 25% thrombocytopenia (four grade 3–4), and 2% had grade 3 febrile neutropenia.

See Supplementary Materials for more details (Table S2).

## 4. Discussion

In the present study, we analyzed a large population of mGBM and reported an incidence of mGBM of 7.2% (98/1354). Other prior studies analyzing mGBM are summarized in Table 5.

The high variability in the incidence of mGBM reported in the literature could probably be due to the small number of cases examined, imaging modality, histologic verification, and selection criteria. However, the literature data suggest that mGBM is not an infrequent entity, reinforcing the concept of the diffusely infiltrative GBM’s nature. Moreover, the incidence will probably increase in the future, due to neuroimaging advances and aging of the population. Some authors have supposed that the higher incidence of mGBM can also be attributed to not yet known environmental factors or genetic modifications that confer higher aggressiveness [23]. In our series, not all patients had a histological diagnosis. These patients were also considered in the analysis because they possessed highly suggestive imaging for GBM, supported also by further investigations such as PET-MRI, PET-TC, and MRI spectroscopy. In addition, gliomas with multiple lesions are more often typically GBMs than lower grade lesions [5]. According to EANO guidelines, histological diagnosis should always be considered unless the risk from biopsy is higher than benefit, like patients with comorbidities, large lesions with typical radiological appearance of GBM, or rapid clinical worsening [24].

Nevertheless, the molecular characterization of mGBM is still controversial. In the study conducted by Dono et al., EGFR mutations and co-occurrence of EGFR/PTEN alteration were statistically significantly more frequently in mGBMs, showing a possible role in its aggressiveness and invasive phenotype [6]. In the analysis of Liu et al., mGBMs were significantly associated with the mesenchymal subtype and the CYB5R2 gene was hypomethylated and overexpressed in mGBMs. Furthermore, the expression level of the CYB5R2 gene was correlated with the OS. CYB5R2 was related to many proteases and toll-like receptor family, indicating a possible role in tumor invasion and immunoregulation. The methylation status of CYB5R2 could be a biomarker for mGBM and a new epigenetic marker for its prognosis [9]. Instead, Patil et al. found no significant differences in the expression profile of MAPK, PTEN, MGMT, laminin β1 and β2, and EGFR between mGBM and unifocal GBM patients [8]. However, this study had a limited sample size and had not an exhaustive molecular characterization. In our study, only IDH1 mutation and MGMT methylation status were considered. MGMT methylated was present in 47.5% of analyzed patients and IDH mutated in no patient. These data are in agreement with those present in the literature. We demonstrated, according to previous studies, that there is no difference in MGMT status between mGBM and unifocal GBM, [4,15]. Rather, IDH mutation in newly diagnosed mGBM is rare because it is usually related to secondary GBM arising from previous lower grade glioma [20,25]. Moreover, according to the new WHO classification, there is no primary GBM IDH-mutated. Liu et al. found that mGBM had no IDH mutation, [9]. Dono et al. found 3% of mGBM as IDH mutant [6] and Lahmi et al. 9% [18]. All these studies refer to older WHO classifications.

In our study, the median overall survival (OS) was 10.2 months (95% CI 6.6–13.8 months) and median progression-free survival (PFS) was 4.2 months (95% CI 3.2–5.2 months). Both values are in line with previously published studies (Table 6). In the literature, the median OS of patients with newly diagnosed mGBM is 6–13 months [6,7,8,9,10,11,12,14,15,18] and the median a PFS is 3.1–6.5 months [7,11,12,18].

However, according to the literature, multifocal presentation at diagnosis seems not to be an independent prognostic factor for OS. Indeed, the worse survival of mGBM is due to the wider tumor dissemination, the more common invasion of eloquent structures and deep areas, and the more frequent presence of an unfavorable PS. Moreover, most mGBM patients cannot be considered a target for a GTR. In the work of Lasocki et al., the difference in survival between mGBM and unifocal GBM patients was not statistically significant in multivariate analysis. Instead, the presence of foci in a deep position or the involvement of the posterior fossa were independent predictors of poor survival, and they were statistically more common in mGBM patients than unifocal GBM ones [14]. Thomas et al. found a survival difference between unifocal, and multifocal/multicentric GBMs. Patients with a single lesion had a median survival of 18 months, ones with multifocal GBM of 10 months, and ones with multicentric GBM of three months, but the difference in survival was no longer significant in multivariate analysis. Conversely, independent predictors of outcome were age, initial KPS score, extent of resection, and MGMT status. However, given that age and MGMT methylation status were comparable between unifocal and multifocal GBMs, the main causes for reduced survival resulted to be the discrepancy in KPS and extent of resection [15].

Indeed, the main positive prognostic factors present in literature and associated with improved survival in mGBM patients are better PS, wider extent of resection (EOR), methylated MGMT promoter, and radiotherapy plus concomitant and adjuvant temozolomide (RT + TMZ) [7,8,10,11,12,13,15,17]. Prognostic factors evaluated in the literature and resulted statistically significant are summarized in Table 7.

In our study, PS was found to be a statistically significant prognostic factor for OS only in the univariate analysis (*p* = 0.012) In contrast to our results, Showalter et al. and Thomas et al. found that the PS was a significant independent prognostic factor for progression and survival in mGBM patients [7,15].

Noteworthy, in the current study, the EOR was not identified as an independent prognostic factor in the multivariate analysis for OS. These findings could be undermined by confounding factors. Indeed, patients selected for surgical resection could have presented with a better PS and/or with fewer comorbidities. Moreover, there is no consensus on the effect of the extent of surgical treatment of mGBM and the survival. Several studies have suggested that the EOR is a prognostic factor for OS [7,8,10,11,13,17], however it was confirmed as an independent prognostic factor only in one study [15]. Maximizing the concept of an extensive resection, Hassaneen et al. evaluated the role of resection of all lesions through multiple craniotomies during a single surgical session. The procedure was associated with a survival rate comparable to that of single craniotomy for a solitary GBM (median OS was 9.7 months vs. 10.5 months, *p* = 0.34), questioning the minimal role of surgery in mGBM [26].

The surgical approach in mGBM is not clear and far from being standardized. Given the advancement of the molecular diagnosis of brain tumors and the progress in targeted therapy, it is mandatory to obtain a histological diagnosis [27]. Moreover, whether PR versus biopsy prolongs survival is not evident, as disclosed in Table 8. Stereotactic biopsy is an efficient and safe method to obtain diagnostic sample. Furthermore, positron emission tomography (PET)-guided stereotactic biopsy could allow the identification of the higher-grade regions of the tumor, resulting in a greater diagnostic yield with lower risk of undergrading of the lesion [28,29,30]. Moreover, achieving a GTR in mGBMs is technically difficult with very few cases reported. Total removal appeared to be possible in selected patients with multiple lesions in non-dominant hemisphere and in non-eloquent regions [8], or in cases with distinct but still close lesions [6,11]. The data relating to the EOR in our study and those present in the literature are summarized in Table 8.

Based on current data, in mGBM patients with good PS and symptomatic localization that could be safely surgically removed, a more aggressive resection, such as gross total or partial resection, might be considered. In other cases, a stereotactic biopsy could be a safer method to obtain the histological diagnosis without decrease the quality of life and survival of patients.

Regarding molecular characterization, in the present study, methylated patients showed a gain in OS over non-methylated patients but without statistical significance; it could be due to limited sample size with several missing data. Particularly in the multivariate analysis for OS, MGMT status had a strong tendency towards statistical significance, suggesting its role as an independent prognostic indicator, as shown in other studies [10,15].

Several factors influenced the choice of active cancer treatment. All patients were discussed at the multidisciplinary neuro-oncology meeting composed of neuro-oncologists, radiotherapists, pathologists, neuroradiologists, neurosurgeons, and neurologists. Generally, in the case of patients with poor PS, unmethylated MGMT the choice turned to radiotherapy alone. In the case of elderly patients, poor PS, methylated MGMT, and extremely widespread disease in many areas, the choice was temozolomide alone.

In our series, the OS benefit from the active oncological treatment was reported only in patients undergoing chemoradiotherapy combination. In fact, the chemoradiotherapy regimen proved to be a strong statistically significant prognostic factor in the univariate analysis for OS and PFS and in the multivariate analysis for OS, finding observed in other studies [10,12,13]. In the work of Haque et al., the analysis was performed in biopsied mGBM patients, demonstrating the role of chemoradiotherapy even in patients without debulking surgery [10]. The OS benefit with the RT + TMZ was about 10 months. Furthermore, the RT + TMZ was associated with the best disease control rate (63%) and with fewer adverse events, most of low grade, demonstrating its efficacy and safety. These good results could be connected to the fact that almost half of the patients were MGMT methylated, which is a predictive factor of treatment response [4,31,32]. Some factors presenting before or during surgical treatment, such as a rapid disease progression, rapid clinical deterioration, onset of seizures, and surgery-related complications, could influence the continuation of active oncological treatment, thus resulting in a reduced survival of these patients.

Recent studies have investigated the role of whole-brain radiotherapy (WBRT) in mGBM. In the work of Lahmi et al., the median OS and PFS of mGBM treated with WBRT in association with concomitant and adjuvant TMZ were 10 months and five months respectively without a severe grade of toxicity [18]. Showalter et al. analyzed the role of WBRT compared to focal three-dimensional conformal radiotherapy (3D-CRT). The latter was associated with a better PFS and OS compared to WBRT. However, 3D-CRT and WBRT had no statistically significant difference in the PFS and OS in the multivariate analysis [7].

## 5. Conclusions

### 5.1. Strengths and Limitations

To the best of our knowledge, this study represents the largest mono-institutional retrospective study on this issue. The study population was selected from a short time frame to reduce the confounding factors regarding changes in clinical practice. The multivariate analysis for OS was carried out to adjust the effect of confounding factors. Nevertheless, several limitations may have impacted the results. The retrospective nature of the study could have led to multiple biases, including selection bias, information bias, and recall bias. Noteworthy, not all patients had a histological diagnosis. Indeed, a quarter of patients (25.5%) had not undergone surgery and the diagnosis of mGBM was supposed on imaging, without the histological certainty. However, physicians used very thorough radiological investigations, such as PET-MRI, PET-TC, and MRI spectroscopy, to achieve a high level of accuracy. Another limitation of the study is that, patients with poor prognoses (comorbidity, elderly, very extensive lesions) have likely received less aggressive therapies, both for surgical and oncological treatments. Another point is the heterogeneity in the molecular evaluation of the histological sample, and it was not available for all patients. The distinction between multifocal and multicentric GBMs was not considered, and the number of lesions and their location have not been evaluated, which could have influenced survival and progression. Furthermore, given the definition of multifocal and multicentric GBM differ between studies, generalization and direct comparison are difficult. Although larger than the other mono-institutional studies in the literature, the sample size is still limited, decreasing the statistical power of the study.

### 5.2. Clinical Implication and Future Perspectives

Glioblastoma remains an incurable disease with poor survival and dismal quality of life, particularly for the subgroup of those with multiple lesions already at the time of diagnosis, representing a challenge in both surgical and oncological approaches. The incidence of mGBM is not rare, and clear therapeutic guidelines are still missing. Patients need to be actively treated and a multidisciplinary approach is mandatory since the treatment alone is trimodal, involving neurosurgeons, radiotherapists, and oncologists. The histological diagnosis should be mandatory, but the surgical approach and the EOR remain unclear. In the coming years, we expect an implementation of the surgical approach to the patient with mGBM thanks to the frameless image-guided stereotactic brain biopsy procedure. Such a technique is executable by more neurosurgeons, and in addition more patients are eligible for it, although not perfectly fit. The results of this study suggest that the active oncological treatment is worth considering. The combination of RT +TMZ is confirmed to be an independent prognostic factor for survival and a treatment with proven efficacy and safety. We suggest that RT + TMZ should be considered as a possible first-line treatment in this subgroup of patients. Prognostic factors such as ECOG PS can help the care teams to define the most appropriate treatments while MGMT methylation status seems not be an important prognostic factor.

Further multicentric studies on this domain with a wider number of patients are required to confirm these results, to clarify the role of surgery, and to improve the survival and quality of life of mGBM patients. Further genetic and epigenetic studies are recommended to identify the invasiveness and aggressiveness of mGBM and to develop new target therapies.

## Figures and Tables

**Table 1:** Patients and treatment characteristics.

Patient Characteristics (*No.* = 98)		
Age—*yr*		
Mean	62	
Median	63	
Range	16–84	
Gender—*no*. (%)		
Male	63	(64.30%)
Female	35	(35.70%)
ECOG PS—*no*. (%)		
0–2	74	(75.50%)
>2	15	(15.30%)
Data missing	9	(9.20%)
Surgery—*no*. (%)		
Yes	73	(74.50%)
No	25	(25.50%)
Extent of resection—*no*. out of 73 (%)		
Biopsy	26	(35.60%)
Partial resection	45	(61.60%)
Gross total resection	0	(0%)
Not otherwise specified	2	(2.70%)
MGMT—*no*. out of 73 (%)		
Data available	59	(80.80%)
Data missing	14	(19.20%)
MGMT status—*no*. out of 59 (%)		
Methylated	28	(47.50%)
Unmethylated	31	(52.50%)
IDH—*no*. out of 73 (%)		
Data available	60	(82.20%)
Data missing	13	(17.80%)
IDH 1–2 status—*no*. out of 60 (%)		
Wild Type	60	(100%)
Mutated	0	(0.00%)
Active oncological treatment—*no*. (%)		
Yes	82	(83.70%)
No	16	(16.30%)
Type of active oncological treatment—*no*. out of 82 (%)		
RT + TMZ	54	(65.90%)
TMZ alone	26	(31.70%)
RT alone	2	(2.40%)
Cycles of temozolomide—*no*.		
Median	2	
Range	0–16	

No = Number; Yr = years; ECOG PS = Eastern Cooperative Oncology Group Performance Status; MGMT = O-6-methylguanine-DNA methyltransferase; IDH = Isocitrate dehydrogenase; RT = Radiotherapy; TMZ = Temozolomide.

**Table 2:** Best response to treatment according to the RANO criteria.

Type of Response—*No.* (%)	Active Oncological Treatment (*No.* = 82)	RT + TMZ (*No.* = 54)	TMZ Alone (*No.* = 26)	RT Alone (*No.* = 2)
CR	0 (0%)	0 (0%)	0 (0%)	0 (0%)
PR	6 (7%)	4 (7%)	2 (8%)	0 (0%)
SD	38 (46%)	30 (56%)	8 (31%)	0 (0%)
ORR (CR + PR)	6 (7%)	4 (7%)	2 (8%)	0 (0%)
DCR (CR + PR + SD)	44 (54%)	34 (63%)	10 (38%)	0 (0%)
PD	38 (46%)	20 (37%)	16 (62%)	2 (100%)

No. = Number; RT = Radiotherapy; TMZ = Temozolomide; CR = Complete Response; PR = Partial Response; SD = Stabile disease; ORR = Overall Response Rate; DCR = Disease Control Rate; PD = Progressive Disease.

**Table 3:** Overall survival (OS) analysis using the Kaplan-Meier method and log-rank test.

	Total *No.*	*No.* of Events	Median OS	IC 95%	*p* Value
Extent of resection					**0.028**
PR	45	31	13.8	6.0–21.6	
Biopsy	26	14	10.2	5.5–14.9	
ECOG PS					**0.012**
0–2	74	49	11.7	9.9–13.5	
>2	15	14	3.5	3.1–3.9	
MGMT status					0.138
Unmet	31	18	12.6	10.0–15.2	
Met	28	19	20.9	6.4–35.4	
Active oncological treatment					**<0.001**
Yes	82	58	11.1	8.2–14.0	
No	16	8	1.9	0.7–3.1	
RT + TMZ					**<0.001**
Yes	54	36	13.8	6.9–20.7	
No	44	30	3.6	2.6–4.7	

No. = Number; ECOG PS = Eastern Cooperative Oncology Group Performance Status; MGMT= O-6-methylguanine DNA methyltransferase; RT = Radiotherapy; TMZ = Temozolomide.

**Table 4:** Multivariate Analysis for OS with Cox regression.

	B	Sig.	HR	IC 95%
ECOG PS		0.07	3	0.9–9.6
>2 vs.	1.08			
0–2	4			
MGMT		0.075	2.1	0.9–5.0
Unmet vs.	0.76			
Met	2			
RT + TMZ		**0.014**	3.1	1.3–7.7
No vs.	1.13			
Yes	6			

ECOG PS = Eastern Cooperative Oncology Group Performance Status; MGMT = O-6-methylguanine DNA methyltransferase; RT = Radiotherapy; TMZ = Temozolomide.

**Table 5:** Incidence of mGBM.

Authors	Incidence of mGBM (%)	*No.* of mGBM Patients
Salvati M. et al., 2003 [5]	2	25
Dono A et al., 2020 [6]	9.9	39
Showalter TN et al., 2007 [7]	10	50
Patil CG et al., 2012 [8]	12.8	47
Liu Q et al., 2015 [9]	15.6	35
Haque W et al., 2020 ^1^ [10]	17.2	7785
Paulsson AK et al., 2014 [11]	21	33
Syed M et al., 2018 [12]	24	63
Kasper J et al. 2021 [13]	29.5	54
Lasocki A et al., 2016 [14]	34	51
Thomas RP et al., 2013 [15]	35	67
Present study	*7.2*	*98*

^1^ This study was the only multicentric retrospective study performed, all the others are mono-institutional retrospective studies.

**Table 6:** The median OS of GBM patients with multiple lesions at the time of diagnosis according to the present study and those in the literature.

Authors	Median OS (Months)
Lasocki A et al., 2016 [14]	6
Patil CG et al., 2012 [8]	6
Liu Q et al., 2015 [9]	6
Showalter TN et al., 2007 [7]	8.1
Paulsson AK et al., 2014 [11]	8.2
Haque W et al., 2020 [10]	8.3
Lahmi L et al., 2019 [18]	10
Thomas RP et al., 2013 [15]	10
Syed M et al., 2018 [12]	11.5
Dono A et al., 2020 [6]	13
Present study	10.2

OS = Overall Survival.

**Table 7:** Significant prognostic factors affecting survival in mGBM patients. Explanations are reported when present.

Authors	Predictive Factors	Explanations
Syed M et al., 2018 [12]	-RT + TMZ (OS univariate, OS multivariate)	NA
Haque W et al., 2020 [10]	-EOR (OS univariate) -RT + TMZ (OS univariate, OS multivariate) -MGMT status (OS univariate, OS multivariate)	EOR: gross total resection vs. subtotal resection vs. biopsy. RT + TMZ: analysis conducted on a subpopulation of biopsied mGBMs. MGMT status: analysis conducted on a population of both multifocal and unifocal glioblastomas.
Patil CG et al., 2012 [8]	-EOR (OS univariate) -KPS (OS univariate)	EOR: gross total or near gross total vs. partial resection or biopsy. KPS: ≥70 vs. <70.
Paulsson AK et al., 2014 [11]	-KPS (PFS univariate)	KPS: ≥80 vs. <80.
Di L et al., 2020 [17]	-EOR (OS univariate, PFS multivariate)	EOR: resection vs. biopsy.
Showalter TN et al., 2007 [7]	-KPS (PFS univariate PFS, PFS multivariate, OS univariate, OS multivariate) -EOR (PFS multivariate, OS univariate) -RT + TMZ (PFS multivariate, OS univariate)	KPS: ≥70 vs. <70 EOG: gross total resection vs. biopsy.
Thomas RP et al., 2013 [15]	-KPS (OS multivariate, PFS multivariate) -EOR (OS multivariate, PFS multivariate) -MGMT status (OS multivariate, PFS multivariate)	EOR: gross total resection vs. less extent of resection.
Kasper J et al., 2021 [13]	-EOR (OS univariate) -RT + TMZ (OS univariate, OS multivariate)	EOR: ratio of residual tumor volume (contrast-enhancing tumor volume assessed from subtraction sequences of T1 MR imaging with and without contrast) and initial tumor volume (sum of contrast-enhancing tumor volume and pre-operative necrosis volume) in percent.
Present study	-PS (OS univariate) -EOR (OS univariate, PFS univariate) -Active treatment (OS univariate, PFS univariate) -RT + TMZ (OS univariate, OS multivariate OS, PFS univariate)	PS: >2 vs. 0–2. EOR: partial resection vs. biopsy. Active treatment: RT + TMZ, TMZ alone, RT alone vs. palliative care.

NA = Not Available; RT = Radiotherapy; TMZ = Temozolomide; EOR = Extend of Resection; MGMT = O-6-methylguanine DNA methyltransferase; KPS = Karnofsy Performance Status; PS = Performance Status; OS = Overall Survival; PFS = Progression Free Survival.

**Table 8:** Data on the extent of resection in mGBM. Explanations are reported when present.

Authors	Surgical Resection %	Explanation
Syed M et al., 2018 [12]	- 14% Total - 38% Subtotal - 48% Biopsy	NA
Dono A et al., 2020 [6]	Multifocal GBM: - 30% Gross total - 0% Near total - 50% Subtotal - 20% Biopsy Multicentric GBM: - 5% Gross total - 5% Near total - 80% Subtotal - 10% Biopsy	NA
Haque W et al., 2020 [10]	- 25.8% Gross total - 35.1% Subtotal - 34% Biopsy - 5.2% Surgery not otherwise specified	NA
Lahmi L et al., 2019 [18]	- 18% Partial - 82% Biopsy	NA
Patil CG et al., 2012 [8]	- 8.5% Gross total - 4.3% Near gross total - 29.8% Partial - 57.4% Biopsy	Gross-total: all of the enhancing tumor resected. Near gross total: >95% of the enhancing tumor resected. Partial: <95% of the enhancing tumor resected. Biopsy: procedure only.
Paulsson AK et al.l, 2014 [11]	Multifocal GBM: - 24% Gross total - 48% Subtotal - 28% Biopsy Multicentric GBM: - 12.5% Gross total - 25% Subtotal - 63.5% Biopsy	Gross total: all enhancing tumor removed. Subtotal: greater than 10% of the tumor mass debulked, but surgery was not a GTR. Biopsy: less than 10% of the tumor was debulked.
Di L et al., 2020 [17]	- 47.1% Resection: - 25% Resection ≥ 85% - 56.3% Resection < 85% - 18.7% Resection not otherwise specified - 52.9% Biopsy	Resection: removal of the largest contrast-enhancing lesion.
Showalter TN et al., 2007 [7]	- 12% Gross total - 66% Subtotal - 22% Biopsy	NA
Thomas RP et al., 2013 [15]	Multifocal - 26% Gross total - 28% Subtotal - 47% Biopsy Multicentric - 0% Gross total - 11% Subtotal - 89% Biopsy	NA
Liu Q et al., 2015 [9]	- 90% Resection - 10% Biopsy	NA
Present study	- 0% Gross total - 61.6% Partial - 35.5% Biopsy - 2.7% Surgery not otherwise specified	Gross total: removal of all enhancing areas on MRI. Partial: ranging from less than 100% of resection to more than 10% of enhancing areas on MRI. Biopsy: resection of less than 10% of the enhancing lesions (open biopsy, stereotactic biopsy).

NA = Not Available; MRI = Magnetic Resonance Imaging.

## Data Availability

All data are available in the text and in the supplementary material.

## Supplementary Materials

The following are available online at https://www.mdpi.com/article/10.3390/curroncol29050280/s1, Figure S1: Progression-free survival of the study population (median PFS = 4.2 months), Figure S2: OS Kaplan-Meier curves for the extent of resection: partial resection (median OS = 13.8 months) vs. biopsy (median OS = 10.2 months), Figure S3: OS Kaplan-Meier curves for the ECOG PS: 0–2 (median OS = 11.7 months) vs. >2 (median OS = 3.5 months), Figure S4: OS Kaplan-Meier curves for the MGMT status: MGMT methylated (median OS = 20.9 months) vs. MGMT unmethylated (median OS = 12.6 months), Figure S5: OS Kaplan-Meier curves for the active oncological treatment: yes (median OS = 11.1 months) vs. no (median OS = 1.9 months), Figure S6: PFS Kaplan-Meier curves for the extent of resection: partial resection (median PFS = 6.6 months) vs. biopsy (median PFS = 3.6 months), Figure S7: PFS Kaplan-Meier curves for the ECOG PS: 0–2 (median PFS = 4.8 months) vs. >2 (median PFS = 3.3 months), Figure S8: PFS Kaplan-Meier curves for the MGMT status: MGMT methylated (median PFS = 5.4 months) vs. MGMT unmethylated (median PFS = 6.8 months), Figure S9: PFS Kaplan-Meier curves for the active oncological treatment: yes (median PFS = 4.8 months) vs. no (median PFS = 1.7 months), Figure S10: PFSS Kaplan-Meier curves for the RT + TMZ: yes (medianOS = 6.9 months) vs. no (median PFS = 2.4 months), Table S1: Progression-free survival (PFS) analysis using the Kaplan-Meier method and log-rank test, Table S2: Adverse events according to the CTCAE v5.0 classification.
