# Supplementary material for: Newly Diagnosed Multifocal GBM: A Monocentric Experience and Literature Review
Source: Curr Oncol. 2022 May 11;29(5):3472–88. doi: 10.3390/curroncol29050280 (PMC9139839; doi:10.3390/curroncol29050280)
Supplement: Supplementary file 1 [file curroncol-29-00280-s001.zip › curroncol-1678620-supplementary.pdf]

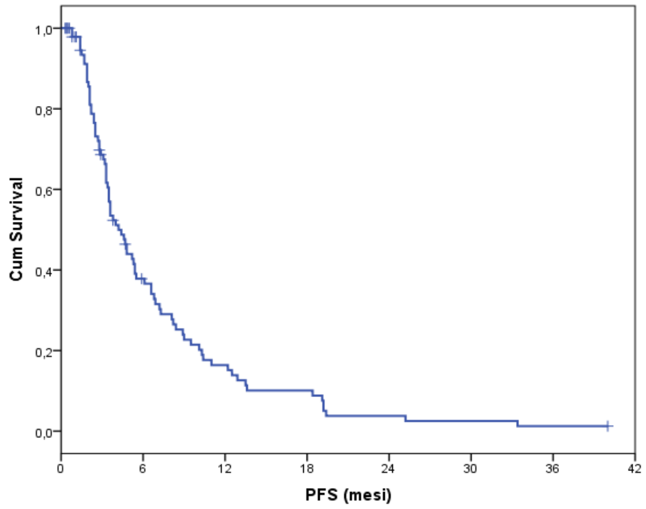

Figure S1. Progression-free survival of the study population (median PFS =4.2 months).

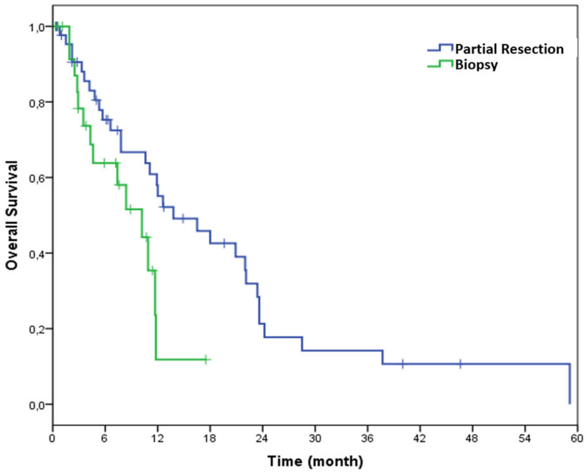

Figure S2. OS Kaplan-Meier curves for the extent of resection: partial resection (median OS=13.8 months) vs biopsy (median OS=10.2 months).

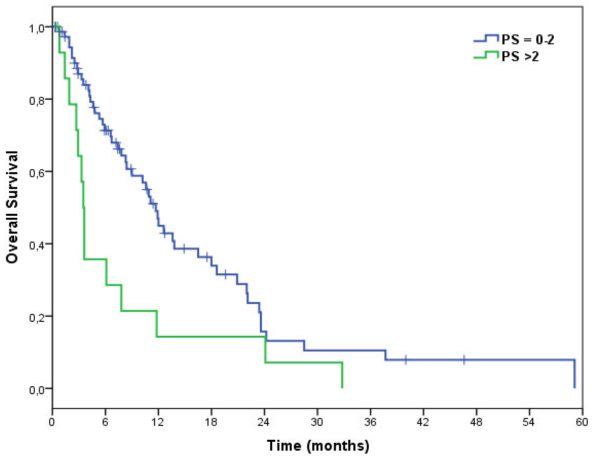

Figure S3. OS Kaplan-Meier curves for the ECOG PS: 0-2 (median OS=11.7 months) vs >2 (median OS=3.5 months).

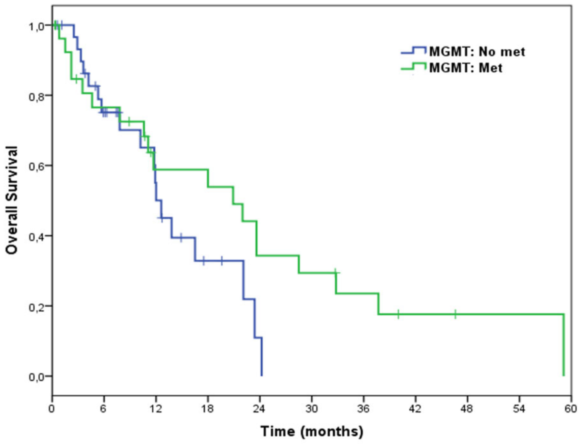

Figure S4. OS Kaplan-Meier curves for the MGMT status: MGMT methylated (median OS=20.9 months) vs MGMT unmethylated (median OS=12.6 months).

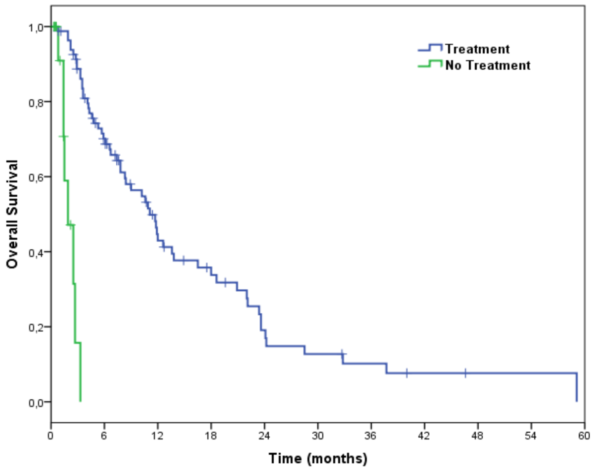

Figure S5. OS Kaplan-Meier curves for the active oncological treatment: yes (median OS=11.1 months) vs no (median OS=1.9 months).

Table S1. Progression-free survival (PFS) analysis using the Kaplan-Meier method and log-rank test.

	Total N	N of Events	Median PFS	IC 95%	P value
Extent of resection					**0.007**
PR	45	40	6.6	5.0-8.2	
Biopsy	26	20	3.6	2.6-4.6	
ECOG PS					0.331
0-2	74	63	4.8	3.5-6.1	
>2	15	14	3.3	2.0-4.6	
MGMT status					0.137
Unmet	31	27	6.8	5.0-8.6	
Met	28	24	5.4	2.2-8.6	
Active oncological treatment					**<0.001**

	Yes	82	74	4.8	3.7-5.9	
	No	16	9	1.7	1.2-2.3	
RT+TMZ						**<0.001**
	Yes	54	50	6.9	4.9-8.9	
	No	44	33	2.4	1.9-2.9	

ECOG PS=Eastern Cooperative Oncology Group Performance Status; MGMT= O-6-methylguanine DNA methyltransferase; RT=Radiotherapy; TMZ=Temozolomide

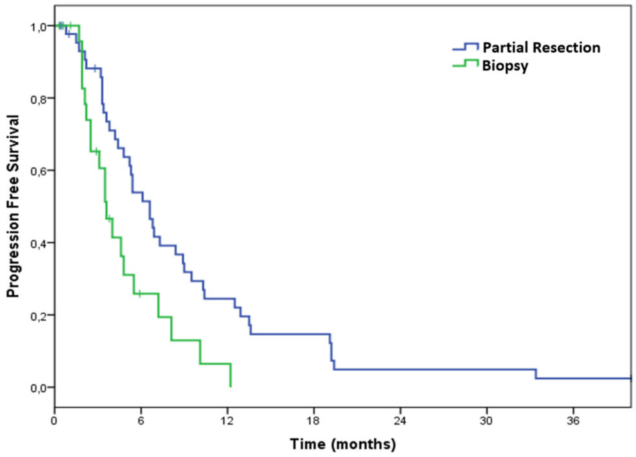

Figure S6. PFS Kaplan-Meier curves for the extent of resection: partial resection (median PFS=6.6 months) vs biopsy (median PFS=3.6 months).

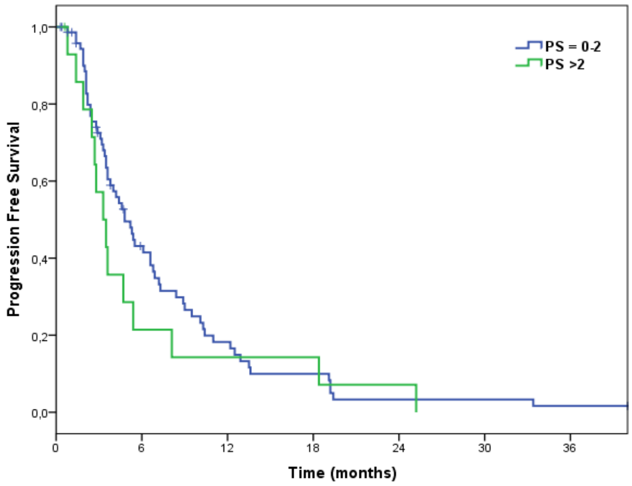

Figure S7. PFS Kaplan-Meier curves for the ECOG PS: 0-2 (median PFS=4.8 months) vs >2 (median PFS=3.3 months).

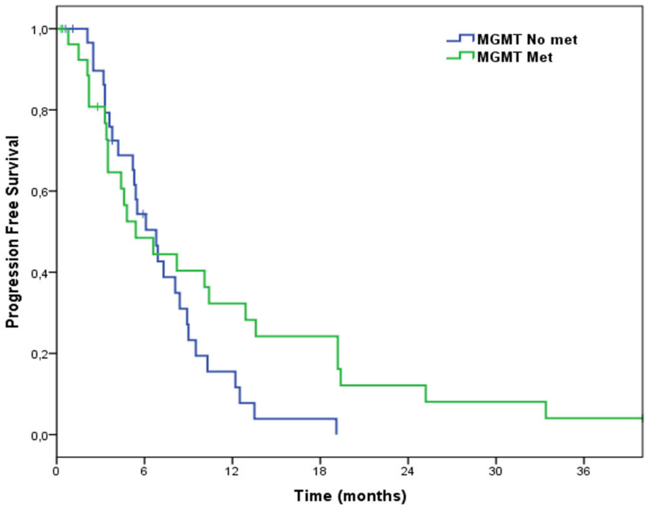

Figure S8. PFS Kaplan-Meier curves for the MGMT status: MGMT methylated (median PFS=5.4 months) vs MGMT unmethylated (median PFS=6.8 months).

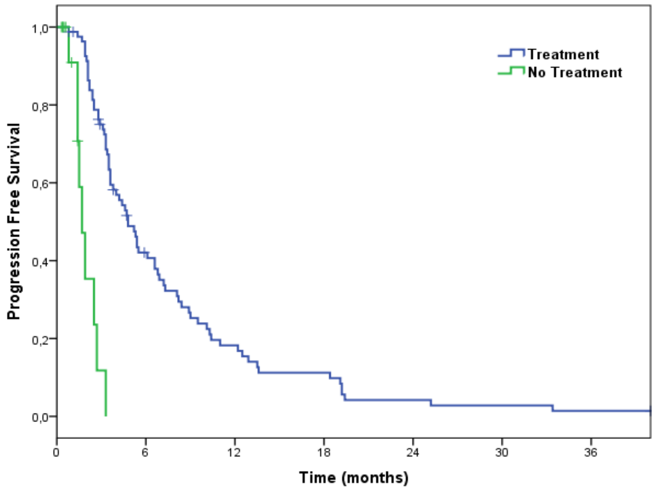

Figure S9. PFS Kaplan-Meier curves for the active oncological treatment: yes (median PFS=4.8 months) vs no (median PFS=1.7 months).

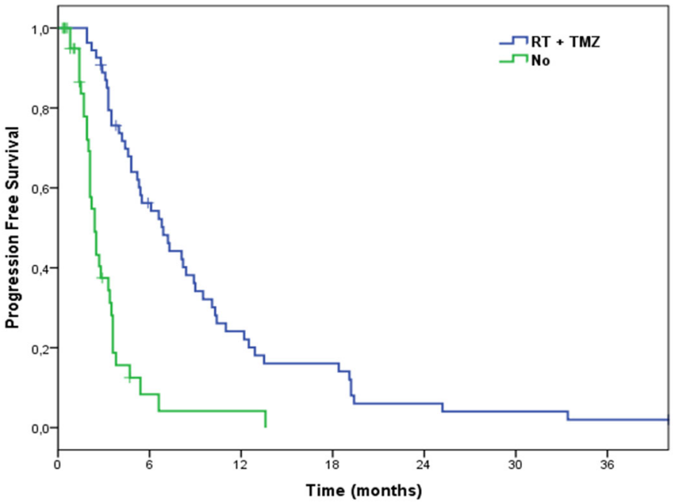

Figure S10. PFSS Kaplan-Meier curves for the RT+TMZ: yes (medianOS=6.9 months) vs no (median PFS=2.4 months).

Table S2. Adverse events according to the CTCAE v5.0 classification.

Adverse events		Active oncol. Treatment (N.82)		RT+TMZ (N.54)		TMZ alone (N.26)		RT alone (N.2)	
**Hematological**									
Anemia				9		5		0	(0%)
	Grade 1	14	(17%)		(17		(19	0	(0%)
	2	2	(2%)	%)		%)		0	(0%)
	3	0	(0%)	1	(2%)	1	(4%)		
	4			0	(0%)	0	(0%)		
		1	(1%)	1	(2%)	0	(0%)	0	(0%)
Neutropenia									
	Grade 1	1	(1%)	1	(2%)	0	(0%)	0	(0%)
	2	1	(1%)	1	(2%)	0	(0%)	0	(0%)
	3	0	(0%)	0	(0%)	0	(0%)	0	(0%)
	4	3	(4%)	1	(2%)	2	(8%)	0	(0%)
Thrombocytopenia				6		9		0	(0%)
	Grade 1	15	(18%)		(11		(35	0	(0%)
	2	3	(4%)	%)		%)		0	(0%)
	3	3	(4%)	3	(6%)	0	(0%)	0	(0%)
	4	3	(4%)	2	(4%)	1	(4%)		
				2	(4%)	1	(4%)		
Febrile Neutropenia									
	Grade 3	1	(1%)	1	(2%)	0	(0%)	0	(0%)
	4	0	(0%)	0	(0%)	0	(0%)	0	(0%)
**Gastrointestinal disorders**									
Nausea									
	Grade 1	4	(5%)	4	(5%)	4	(5%)	0	(0%)
	2	1	(1%)	1	(1%)	1	(1%)	0	(0%)
	3	0	(0%)	0	(0%)	0	(0%)	0	(0%)
Constipation						4		0	(0%)
	Grade 1	5	(6%)	1	(2%)		(15	0	(0%)
	2	0	(0%)	0	(0%)	%)		0	(0%)
	3	1	(1%)	1	(2%)	0	(0%)	0	(0%)
	4	0	(0%)	0	(0%)	0	(0%)		
						0	(0%)		
AST/ALT elevation									
	Grade 1	6	(7%)	4	(7%)	2	(8%)	0	(0%)
	2	1	(1%)	0	(0%)	1	(4%)	0	(0%)
	3	0	(0%)	0	(0%)	0	(0%)	0	(0%)
	4	0	(0%)	0	(0%)	0	(0%)	0	(0%)
GGT elevation									
	Grade 1	0	(0%)	0	(0%)	0	(0%)	0	(0%)
	2	0	(0%)	0	(0%)	0	(0%)	0	(0%)
	3	1	(1%)	0	(0%)	1	(4%)	0	(0%)
	4	0	(0%)	0	(0%)	0	(0%)	0	(0%)
Hyperbilirubinemia									
	Grade 1	1	(1%)	1	(2%)	0	(0%)	0	(0%)
	2	0	(0%)	0	(0%)	0	(0%)	0	(0%)
	3	0	(0%)	0	(0%)	0	(0%)	0	(0%)
	4	0	(0%)	0	(0%)	0	(0%)	0	(0%)

**General disorders**									
Fatigue					7			0	(0%)
	Grade 1	9	(11%)		(13	2	(8%)	0	(0%)
	2	3	(4%)		%)	1	(4%)	0	(0%)
	3	0	(0%)	2	(4%)	0	(0%)		
				0	(0%)				
**Infections**									
Herpes zoster		1	(1%)	1	(2%)	0	(0%)	0	(0%)

RT=Temozolomide; RT=Radiotherapy; AST=Aspartate transaminase; ALT=Alanine transaminase; GGT=Gamma-glutamyl transferase.
